# *Bacillus* Species: Excellent Biocontrol Agents against Tomato Diseases

**DOI:** 10.3390/microorganisms12030457

**Published:** 2024-02-24

**Authors:** Vasiljka Karačić, Dragana Miljaković, Jelena Marinković, Maja Ignjatov, Dragana Milošević, Gordana Tamindžić, Milan Ivanović

**Affiliations:** 1 Faculty of Agriculture, University of Belgrade, Zemun, 11080 Belgrade, Serbia; vasiljka.dragic@gmail.com (V.K.); milanivanovic@agrif.bg.ac.rs (M.I.); 2 Institute of the Field and Vegetable Crops, 21000 Novi Sad, Serbia; jelena.marinkovic@ifvcns.ns.ac.rs (J.M.); maja.ignjatov@ifvcns.ns.ac.rs (M.I.); dragana.milosevic@ifvcns.ns.ac.rs (D.M.); gordana.tamindzic@ifvcns.ns.ac.rs (G.T.)

**Keywords:** *Bacillus*, biocontrol mechanisms, disease management, tomato pathogens

## Abstract

Tomatoes encounter many pathogens, such as fungi and bacteria, which reduce the yield and quality of plants and lead to large losses in production. The application of plant protection products (PPPs) is still an important and most effective measure to control plant diseases. However, the use of chemicals in agriculture contributes to environmental pollution and biodiversity loss, and it can also threaten non-target living organisms. Biological control is a widely accessible, environmentally safe, and cost-efficient alternative for the prevention and suppression of plant diseases. *Bacillus* species with antimicrobial and plant growth-promoting effects are most frequently used as biocontrol agents to increase the resilience of agricultural production against biotic stresses. The present review discusses the antagonistic mechanisms and the biocontrol potential of *Bacillus* spp. against tomato diseases caused by different pathogens. The main mechanisms of *Bacillus* spp. include the production of antimicrobial compounds (antibiotics, extracellular enzymes, siderophores, and volatile compounds), competition for nutrients and space, and induced systemic resistance (ISR). Although *Bacillus*-based PPPs have been developed and commercialised worldwide for various crops and pathogens, the efficiency issues are still subject to debate. Additionally, a combined strategy for controlling tomato diseases based on *Bacillus* spp. and other available methods (conventional or natural-based) is a promising research field.

## 1. Introduction

Tomato (*Solanum lycopersicum* L., Solanales: Solanaceae) is one of the most important vegetable crops in the world, cultivated on 5.17 million hectares with a total production of 189 million tons and an average yield of 36.6 tons per hectare [1]. Tomatoes can be grown in a wide area from 55° north to 35° south latitude, with the largest producers being China, India, the USA, Turkey, Egypt, Italy, Russia, and Mexico. They are widely used for fresh or processed consumption due to the presence of important nutrients and bioactive compounds with well-established health benefits [2].

Tomatoes can be infected by more than two hundred different pathogens during the growing and post-harvest periods [3]. The main causative agents of tomato diseases are phytopathogenic fungi and bacteria, which affect both the quality and quantity of tomato production [4,5]. The most important fungi that infect tomato are *Alternaria solani* Sorauer (Pleosporales: Pleosporaceae), *Septoria lycopersici* Spegazzini (Mycosphaerellales: Mycosphaerellaceae), *Botrytis cinerea* Persoon (Helotiales: Sclerotiniaceae), *Fusarium oxysporum* f. sp. *lycopersici* (Saccardo) Snyder and Hansen (Hypocreales: Nectriaceae), *F. oxysporum* f. sp. *radicis*-*lycopersici* Jarvis and Shoemaker (Hypocreales: Nectriaceae), *Verticillium dahliae* Klebahn (Glomerellales: Plectosphaerellaceae), and *Phytophthora infestans* (Montagne) de Bary (Peronosporales: Peronosporaceae) [3]. The major bacterial diseases of tomato are caused by *Pseudomonas syringae* pv. *tomato* (Okabe) Young, Dye and Wilkie (Pseudomonadales: Pseudomonadaceae); *Clavibacter michiganensis* subsp. *michiganensis* (Smith) Davis et al. (Micrococcales: Microbacteriaceae); *Xanthomonas campestris* pv. *vesicatoria* (Doidge) Vauterin, Hoste, Kersters and Swings (Lysobacterales: Lysobacteraceae); and *Ralstonia solanacearum* (Smith) Yabuuchi et al. emend. Safni et al. (Burkholderiales: Burkholderiaceae) [6,7].

Various management strategies, including resistant tomato cultivars as well as cultural, physical, chemical, and biological methods, have been employed globally to control tomato diseases [8]. The application of plant protection products (PPPs) is still a significant method to achieve effective pathogen control and prevent yield losses [9]. However, the excessive usage of PPPs has led to the pollution of surface and underground water, degradation of soil, a negative impact on non-target organisms, and the emergence of pathogen resistance [10]. Public concern about residues in vegetables has increased demand for more precise and strict regulations regarding the use of PPPs. Biological control is one of the most promising alternatives to chemical control of plant diseases, being of particular importance in protected and organic vegetable production [11]. 

The species of the genus *Bacillus* are one of the most studied and used agents in biological control [12]. *Bacillus* spp. demonstrate great antimicrobial activity against numerous pathogens, along with stimulating effects on plant growth and yield [13]. Several *Bacillus* spp. have been proven to be promising biocontrol agents for controlling tomato pathogens, both in laboratory and field conditions [14,15,16].

This review summarises the most important tomato diseases and pathogens; *Bacillus* spp. used as antagonists in tomatoes and their mechanisms of action; and combined management strategies involving *Bacillus* spp. against tomato disease-causing agents.

## 2. Tomato Diseases and Pathogens

Numerous plant diseases lead to large losses in tomato production in both greenhouse and field conditions [17]. Additionally, tomatoes can be infected during harvesting, postharvest, and storage, so they must be used in a timely manner [18]. Tomato diseases can be caused by a wide range of plant pathogens, including fungi, bacteria, viruses, oomycetes, viroids, and phytoplasmas, as well as pests such as nematodes, insects, and mites. However, it is reported that more than 50% of tomato diseases and major yield losses are caused by fungal pathogens [19]. The most important tomato diseases are shown in Table 1.

**Figure 1:**
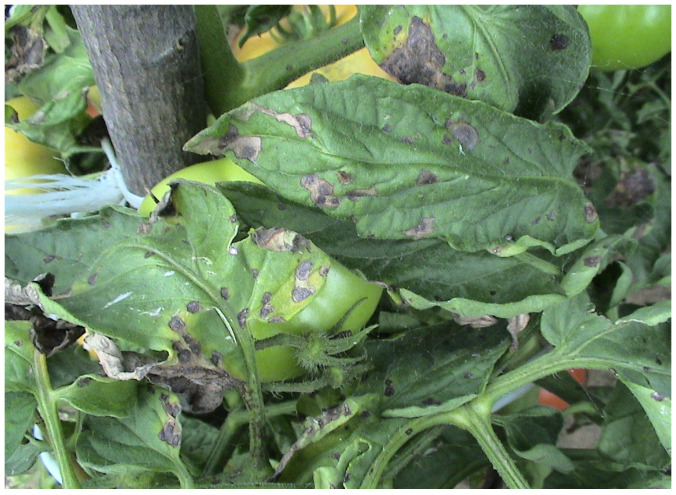
Early blight symptoms on tomato leaves (M. Ivanović).

The fungus *A. solani* is among the most destructive pathogens affecting tomatoes. It causes an early blight disease that is responsible for fruit yield losses ranging from 35 to 78% [20]. Favorable conditions for the spread of *A. solani* include high humidity, frequent rainfall, and temperatures between 24 °C and 29 °C [21]. Symptoms of early blight begin on young leaves as small black-brownish lesions that enlarge and form target-like concentric rings (Figure 1). The lesions spread and lead to the loss of photosynthetic tissue, which ultimately results in damaged fruits covered with a black spore mass. In addition to the leaves and fruits, this pathogen infects the stem and branches, affecting the entire growth of tomato plants [22].

**Figure 2:**
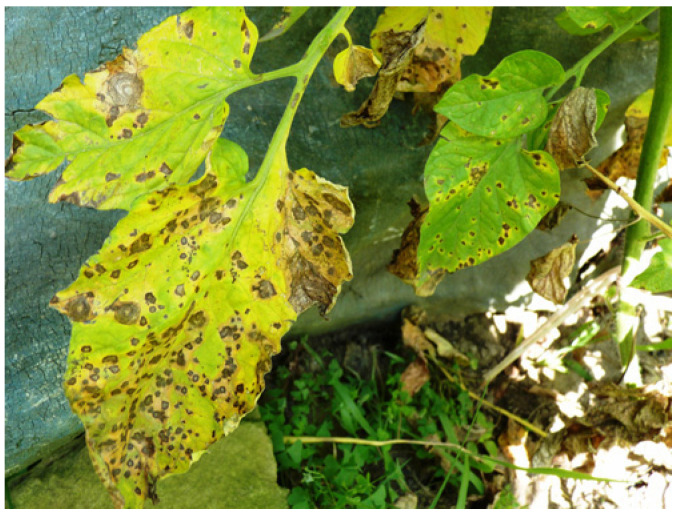
Septoria leaf spot on tomato leaves (P. Vukša).

Septoria leaf spot, caused by *S. lycopersici*, is a significant foliar disease affecting tomatoes worldwide (Figure 2). Yield losses caused by this pathogen are mainly associated with reduced photosynthetic activity and plant growth, as well as the formation of low-quality fruits. In periods when temperatures are above 25 °C in combination with heavy rainfall, especially in the summer, yields can be reduced by more than 50% [23]. Symptoms appear on the leaves in the form of circular, elliptical necrotic lesions with brown to grey centres [24]. Mani et al. [25] point out that the leaf spot disease causes enormous damage in tomato plants at any stage of plant development by entering through the stomata or penetrating the epidermis.

**Figure 3:**
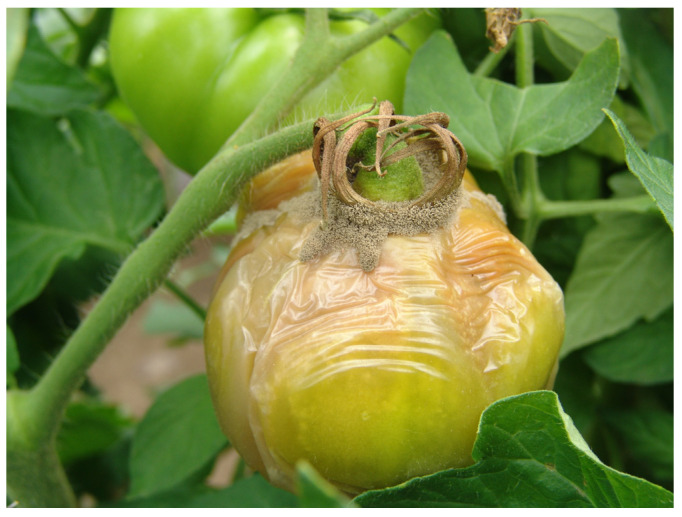
Grey mould symptoms on tomato fruits (M. Ivanović).

The fungus *B. cinerea* is a polyphagous pathogen responsible for serious economic losses in tomatoes [26]. Grey mould can cause damage to all above-ground plant plants in the open field and greenhouse, but also during the transportation and storage of products [27]. Disease is favoured by high humidity and cool temperatures from 18 °C to 23 °C. Infection begins with the appearance of irregular and V-shaped brown blotches on leaves, followed by brown and oval lesions on stems and pale or white rings on fruits (Figure 3). The sensitivity to fungal infection changes with tissue development and ageing, while green fruits are more resistant than red tomato fruits. During development in plant tissue, *B. cinerea* produces toxins that cause plant cells to lose their function [28].

Fusarium vascular wilt, caused by *F. oxysporum* f. sp. *lycopersici*, is one of the destructive diseases of tomatoes that occurs both in the field and greenhouse. It causes yield losses of 45–55%, and in favourable conditions, when temperatures are 27–30 °C, losses can be up to 70% [3]. Srinivas et al. [29] reported that *Fusarium* vascular wilt diseases of tomatoes can reduce the yield of tomatoes to the maximum. This disease blocks xylem and, therefore, water transport [30]. The disease is characterised by wilted plants with yellowed leaves, while the root becomes necrotic and also changes the colour of the vascular tissue [31]. *F. oxysporum* f. sp. *lycopersici* is spread through irrigation water and planting material, while in contaminated soil it can survive for decades [8].

Another very important phytopathogenic fungus that is transmitted in the soil is *F. oxysporum* f. sp. *radicis-lycopersici*. It causes Fusarium crown and root rot in tomatoes and leads to significant yield losses [32]. Panno et al. [3] state that the loss of yield caused by this pathogen can be up to 90% in cases when the weather is cold (<20 °C), but even at high temperatures (27 °C), the occurrence of the disease on tomato plants has been recorded. Unlike *F. oxysporum* f. sp. *lycopersici*, which moves through the xylem, *F. oxysporum* f. sp. *radicis-lycopersici* begins colonisation in areas where the root grows and moves towards the crown of the root, where it blocks the vessels by producing enzymes and toxins [33]. This leads to the wilting and death of the plants. It can survive in the soil for a long time, and once it is introduced into the field, it is almost impossible to eliminate [32].

*V. dahliae* is a fungal pathogen that causes Verticillium wilt of tomatoes [34]. Yield reduction can be 20–50%, especially when optimal temperatures for the growth of *V. dahliae* are present (21–30 °C) [35]. The pathogen can remain dormant in the soil or on dead plants for a long time. When hyphae adhere, they penetrate the roots of plants and prevent the transport of nutrients. Because of this, symptoms such as foliar chlorosis, wilting, stunting, and necrosis appear [36]. Through the tips of the roots, or lateral roots, the pathogen attacks the plant and spreads from the xylem to the aerial part of the plant [37]. 

**Figure 4:**
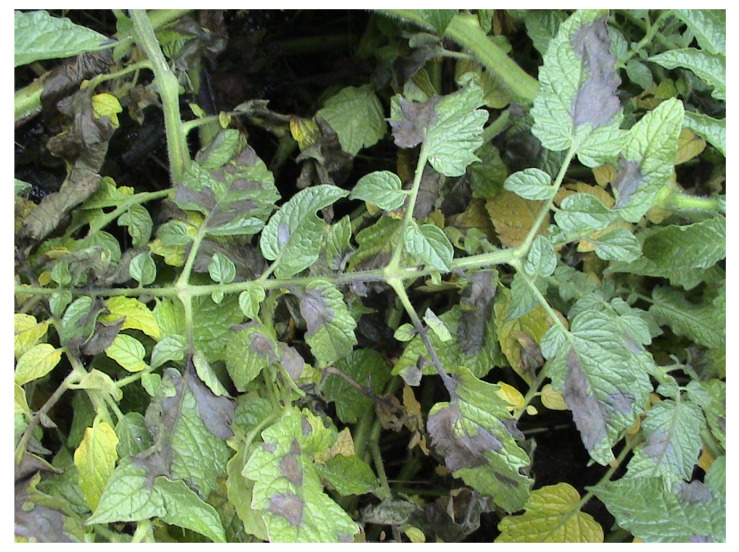
Late blight symptoms on tomato leaves (M. Ivanović).

Late blight, caused by the oomycete *P. infestans*, is one of the most devastating tomato diseases. Under favourable conditions for the pathogen, such as high humidity and temperature, *P. infestans* can destroy the entire tomato production [38]. Symptoms that appear in the field are usually dark grey to brown spots on leaf tissues (Figure 4). In conditions of high humidity, with more than 90%, and a low temperature of 10–20 °C, the infection spreads very quickly. Complete necrosis in the entire field can occur after 5–10 days [39]. Maxim et al. [40] pointed out that finding varieties resistant to *P. infestans* is essential in tomato production, which will reduce the use of fungicides.

The phytopathogenic bacterium *P. syringae* pv. *tomato* causes bacterial speck on tomatoes. Temperatures between 18 °C and 25 °C, followed by high humidity, favour the development of bacterial speck [41]. This disease can cause a yield loss of 75% in cases of early infection [42]. The bacterium can be transmitted via infected seeds and spread over long distances by wind and rain [43]. Due to its great economic importance, *P. syringae* pv. *tomato* is a quarantine bacterium in many countries [44]. In the epiphytic phase, the bacterium adheres to the leaves and creates spots, but the plants do not necessarily die, while in the endophytic phase, the pathogen penetrates into the tissue of the leaves and causes the death of the plants [45]. In addition to the leaves, the symptoms also appear on the stems and fruits in the form of dark spots, which affect the quality and yield of fruits.

**Figure 5:**
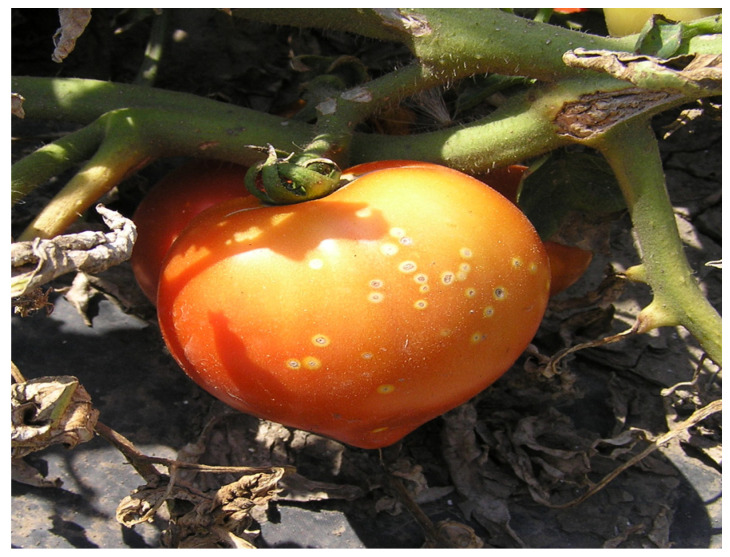
Bacterial wilt and canker symptoms on tomato fruit (S. Milijašević-Marčić).

The bacterium *C. michiganensis* subsp. *michiganensis* causes bacterial wilt and canker in tomatoes, leading to severe economic losses in production worldwide. Wang et al. [46] pointed out that depending on the method of cultivation, location, genotype, and physiological stage of the host, yield losses range from 10% to 100%. The disease develops fastest in young tomato plants with a temperature range of 25 °C to 28 °C and high humidity [47]. The European and Mediterranean Plant Protection Organization (EPPO) characterised this bacterium as a quarantine pathogen. Disease is transmitted by seeds and spread over long distances by rain, irrigation, and other cultural practices [48]. This bacterium colonises plants and reaches the vascular system. The symptoms it causes on the plants depend on the age of the tomatoes and the environmental conditions, but mainly involve mealy spots on the stems, yellowing or wilting of the leaves, and characteristic spots on the fruits known as “bird’s eye” (Figure 5) [49].

**Figure 6:**
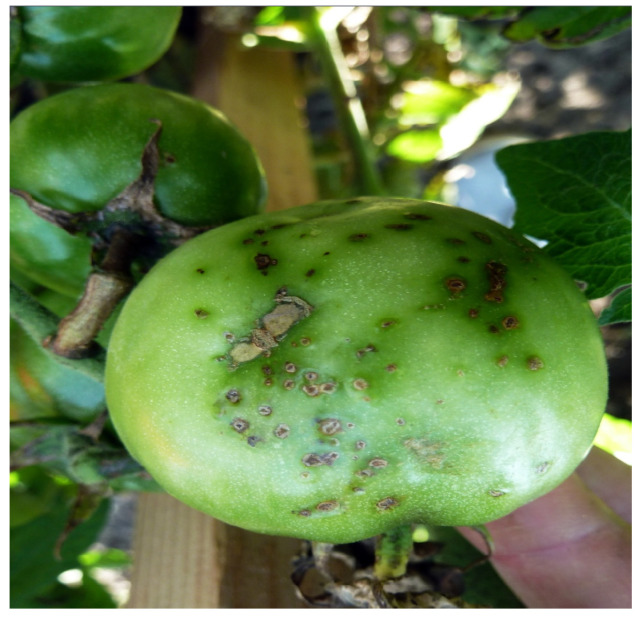
Bacterial spot symptoms on tomato fruit (M. Ignjatov).

Bacterial spot of tomato is caused by bacteria from the *Xanthomonas* group. These bacteria can cause serious damage both in greenhouses and in the field, triggering yield losses ranging from 10% to 50% [50]. The *Xanthomonas* group includes four pathogens, namely, *Xanthomonas euvesicatoria* pv. *euvesicatoria*, *X. euvesicatoria* pv. *perforans*, *X. vesicatoria*, and *X. hortorum* pv. *gardneri* [51]. Symptoms of bacterial spots include black, chlorotic spots on leaves, stems, petioles, and fruits; defoliation; and even scab-like lesions on fruits (Figure 6) [52]. 

Another significant causative agent of bacterial wilt of tomato is the soil-borne bacterium *R. solanacearum* [53]. Mekonnen et al. [54] reported that depending on *R. solanacearum* strains, soil type, host variety, and environment, yield losses in tomatoes can range from 10% to 100%. The *Ralstonia* group includes three pathogens: *Ralstonia pseudosolanacearum*, *R. solanacearum*, and *R. sygzii* [55]. These bacteria colonise xylem tissue, infect the roots of tomato plants, and produce an exopolysaccharide that creates a blockage in the xylem and causes the wilting, yellowing, or stunting of plants [56].

**Table 1:** The economically important tomato diseases caused by phytopathogenic fungi, oomycetes, and bacteria.

Disease	Causative Agent	Symptoms	Epidemiology	Reference
Early blight	*Alternaria solani*	Black-brown, concentric lesions on the leaves, stems, and fruits; yellowing.	It survives on plant debris, seeds, volunteer Solanaceous crops, and soil; favoured by high humidity and temperatures of 24–29 °C.	[22]
Septoria leaf spot	*Septoria lycopersici*	Tan-to-grey spots with dark margins on the leaves.	It survives on plant debris, seeds, volunteer Solanaceous crops, and weeds; favoured by high humidity and temperatures above 25 °C.	[23]
Grey mould	*Botrytis cinerea*	Brown lesions on the leaves and stems; pale or white rings on the fruits.	It survives on plant debris, seeds, soil, and various hosts; favoured by high humidity and temperatures of 18–23 °C.	[28]
Fusarium vascular wilt	*Fusarium oxysporum* f. sp. *lycopersici*	Yellowing; wilting; browning; stunted growth.	It survives in the soil and on plant debris, seeds, and seedlings; favoured by high humidity and temperatures of 20–30 °C.	[29]
Fusarium crown and root rot	*F. oxysporum* f. sp. *radicis*-*lycopersici*	Yellowing; wilting; browning; stunted growth; stem and root discoloration.	It survives in the soil and on plant debris, seeds, and seedlings; favoured by high humidity and temperatures of 10–20 °C.	[33]
Verticillium wilt	*Verticillium dahliae*	Yellowing; wilting; stunted growth; v-shaped lesions on the leaves; stem discoloration.	It survives in the soil and on plant debris; favoured by high humidity and temperatures of 21–30 °C.	[35]
Late blight	*Phytophthora infestans*	Green-black lesions on the leaves; dark spots on the fruits.	It survives on plants, tubers, soil, seeds, Solanaceous crops, and weeds; favoured by high humidity and temperatures of 10–20 °C.	[39]
Bacterial speck	*Pseudomonas syringae* pv. *tomato*	Brown-black spots on the leaves; stunted growth; dark specks on the fruits.	It survives on plant debris, soil, seeds, and weeds; favoured by high humidity and temperatures of 13–25 °C.	[45]
Bacterial wilt and canker	*Clavibacter michiganensis* subsp. *michiganensis*	Wilting; yellowing; stunted growth; stem discoloration; white spots with a necrotic centre on the fruits (bird’s eye).	It survives on plant debris, soil, seeds, and weeds; favoured by high humidity and temperatures of 24–28 °C.	[48]
Bacterial spot	*Xanthomonas campestris* pv. *vesicatoria*	Elliptical, dark, chlorotic spots on the leaves, stems, and fruits.	It survives on plant debris, soil, and seeds; favoured by high humidity and temperatures of 23–30 °C.	[50]
Bacterial wilt	*Ralstonia solanacearum*	Wilting; root rot; stem discoloration and decay.	It survives on plant debris, soil, seeds, seedlings, and weeds; favoured by high humidity and temperatures above 29 °C.	[53]

Tomato disease control involves preventive cultural measures such as the use of certified seeds, healthy transplants, crop rotation, proper plant density, weed control, eradication of volunteer solanaceous crops, adequate nutrition, destruction of infected plant parts, removal of plant residues, drip irrigation, and selection of resistant tomato varieties [57]. Physical measures such as soil solarisation, soil heating, and seed heating may also be used for the tomato disease control [58]. Furthermore, the most common and effective method for tomato disease control is the use of plant protection products (PPPs). There is a wide range of PPPs available to control phytopathogenic fungi and bacteria on tomatoes. The excessive use of fungicides and bactericides has caused soil pollution, a reduction of the microbial population in the soil, and the occurrence of resistant pathogens [59]. Also, the continuous application of agrochemicals negatively affects the nutritional content and bioactive compounds of tomatoes, as well as the structure and productivity of the soil [60]. 

In order to minimise detrimental effects on the environment and public health and ensure food security, both scientists and growers must prioritise the search for more ecologically friendly disease control strategies [61]. The term biological control refers to the use of antagonistic microorganisms, i.e., biological control agents (BCAs), for plant disease control [62]. According to O’Brien [63], the most common biocontrol agents are bacterial or fungal antagonistic strains, isolated from the rhizosphere or endosphere. Biocontrol agents have recently been excluded from the term bioprotectants, which involve the use of extracted or fermented non-living natural products for disease management [64]. Biological agents have found application in conventional, organic, and integrated production of various field and vegetable crops, including tomatoes [65]. The bacteria of the genus *Bacillus* are the predominant biocontrol agents, with the *B. subtilis* complex being the most widely used for controlling plant diseases [66,67].

## 3. *Bacillus* Species: General Characteristics and Benefits of Application

The genus *Bacillus* represents a heterogeneous group of bacteria that are Gram- and catalase-positive, motile, aerobic, or facultatively anaerobic. These bacteria are rod-shaped, straight, and large, being 0.5–2.5 μm wide and 1.2–10 μm long [68]. They use a wide range of carbon sources for heterotrophic or autotrophic growth, showing great metabolic diversity. Bacilli produce dormant endospores, which allow them to survive in adverse environments [69]. Endospores may be central or terminal in the cell, while the cells occur singly, in pairs, or in chains. Due to their ability to grow and sporulate in a wide range of pH values, temperatures, and salinity levels, *Bacillus* spp. are ubiquitous in diverse natural habitats, including soil and plants [70]. Furthermore, *Bacillus* spp. produce biofilm, which also contributes to their colonisation, survival, adaptability, application, and effectiveness [71].

**Figure 7:**
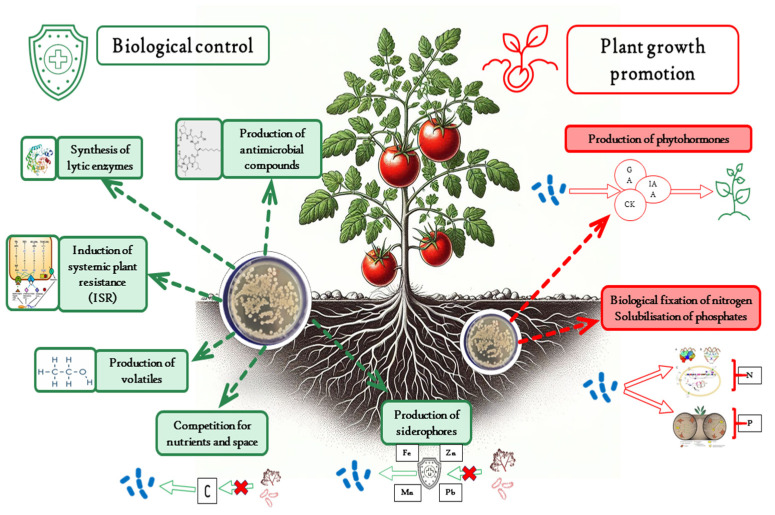
Mechanisms of action of *Bacillus* biocontrol and plant growth-promoting agents.

Only a few species of this genus are pathogenic, whereas others have a predominately positive effect on the growth and yield of plants [13]. Beneficial *Bacillus* species have broad implementation in agriculture for their favourable culturing characteristics and superior production of diverse bioactive compounds [72]. The *Bacillus* most commonly used in agriculture is *Bacillus thuringiensis* due to its insecticidal properties, which are valuable in the biological fight against phytophagous insects. The focus of this review is on the species of the *Bacillus subtilis* complex, such as *B. subtilis*, *B. amyloliquefaciens*, *B. velezensis*, *B. licheniformis*, *B. mojavensis*, *B. pumilus*, and others, that are mostly used against pathogens [73]. Furthermore, these bacteria are generally recognised as safe (GRAS). Biocontrol mechanisms by which *Bacillus* spp. protect plants from pathogens include antibiotic production, synthesis of lytic enzymes, competition for nutrients and space, production of siderophores, production of volatile compounds, and induced systemic plant resistance (ISR) [74]. Moreover, *Bacillus* spp. stimulate plant growth by producing phytohormones such as indole-3-acetic acid (IAA), gibberellins (GA), and cytokinins (CK) [75]. Additionally, they produce hormones that are important in regulating plant stress responses, such as abscisic acid (ABA), salicylic acid (SA), and jasmonic acid (JA) [76]. Certain *Bacillus* spp. produce ACC deaminase, which degrades 1-aminocyclopropane-1-carboxylic acid (ACC) and thus modulates ethylene concentration in plants under stress [77]. *Bacillus* spp. also influence the availability of nutrients in the soil and promote plant growth via nitrogen fixation, phosphate solubilisation, and the production of siderophores [78,79]. Diversity of their mechanisms of action allows them to simultaneously protect the host plant from pathogen infection while stimulating plant growth (Figure 7).

## 4. Mechanisms of Biological Control

### 4.1. Antimicrobial Compounds 

**Figure 8:**
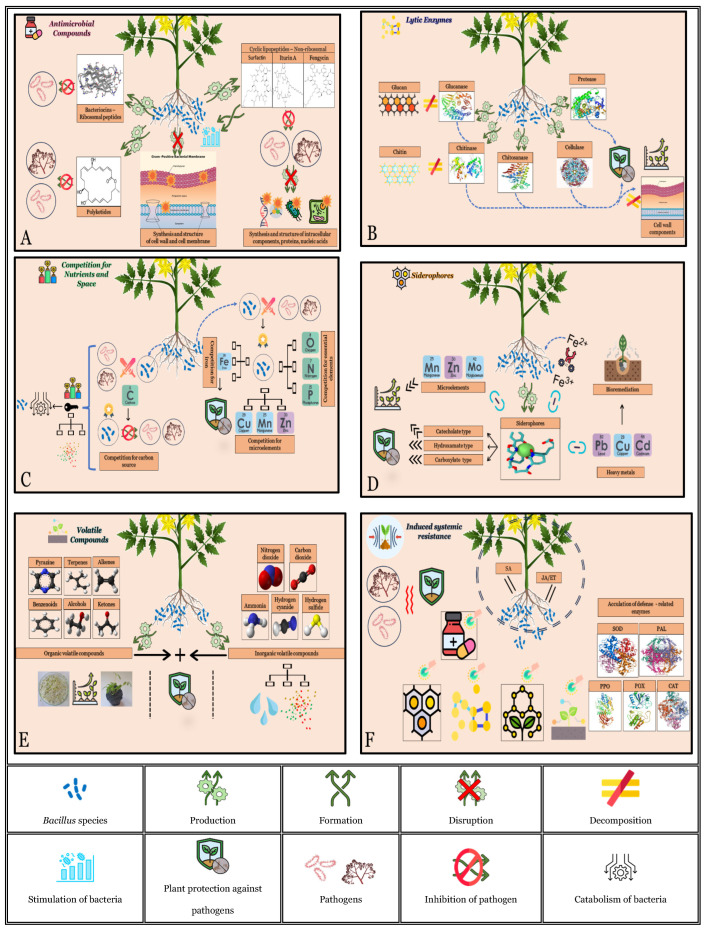

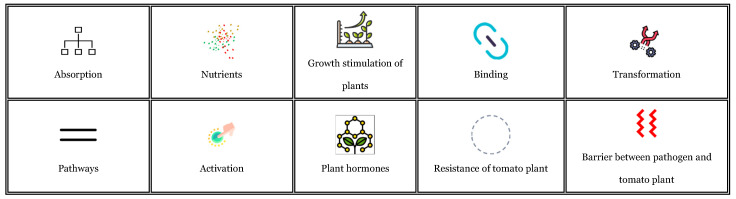
Biocontrol mechanisms of *Bacillus* species. (**A**) Antimicrobial compounds. (**B**) Lytic enzymes. (**C**) Competition for nutrients and space. (**D**) Siderophores. (**E**) Volatile compounds. (**F**) Induced resistance.

Antimicrobial compounds (AMCs) are secondary metabolites that belong to heterogeneous groups of organic compounds produced by microorganisms (Figure 8). The number of known antibiotics produced by actinomycetes (8700), bacteria (2900), and fungi (4900) is enormous [80]. Bacteria from the genus *Bacillus* produce various secondary metabolites that mediate antibiosis, devoting 5–8% of the total genome to their biosynthesis [81]. Based on biosynthetic pathways, antimicrobial compounds are classified into three groups, namely, ribosomal peptides (RPs), non-ribosomal lipopeptides and peptides (NRPs), and polyketides (PKs) [82]. Bacteriocins belong to the RP group and exhibit a broad spectrum of inhibitory activities against closely related bacteria [82]. Most bacteriocins act by destroying the cell wall or disrupting the cytoplasmic membrane [83]. They provide an advantage in competitive bacterial surroundings, especially against multidrug-resistant bacteria [84]. Bacteriocins and bacteriocin-like substances, including amylolysin, amisin, subtilin, subtilosin A, subtilosin B, thuricin, entianin, and ericin, have been isolated from different *Bacillus* spp. [81,82]. However, there is a lack of data on the effect of *Bacillus*-produced bacteriocins against tomato bacterial diseases.

Cyclic lipopeptides (LPs) form a large class of NRP antibiotics that exert their action against a multitude of bacterial and fungal pathogens. The antibacterial activity of LPs works by attaching to the cell membrane of target bacteria, causing perforations and ion leakage, followed by rapid depolarisation and inhibition of DNA, RNA, and protein synthesis, and finally cell death [85]. Antifungal LPs primarily exhibit their action on chitin and (1–3)-β-D-glucan synthases, thereby disrupting cell wall synthesis, osmotic pressure regulation, and the entire cell morphology of pathogenic fungi [86]. They also influence the synthesis and maintenance of other cellular structures, such as the cell membrane and intracellular components (e.g., proteins, nucleotides, mitochondrial membranes, nucleus, and endoplasmic reticulum) [86]. Besides their antimicrobial actions, LPs are important for bacterial motility utilised in growth, reproduction, survival, competition, and colonisation; biofilm formation in the context of its promotion or inhibition; and heavy metal removal from the polluted environment [87]. In *Bacillus* species, the presence of three main LPs families has been confirmed, i.e., surfactin (e.g., surfactin, lichenisin, pumilacidin, and halobacillin), iturin (e.g., iturin A, bacillomycin L, bacillomycin D, bacillomycin F, and mycosubtilin), and fengycin (e.g., fengycin, plipastatin, and maltacin) [88]. Surfactins show antifungal and antibacterial activity, whereas iturins and fengycins are predominately antifungal compounds [89]. 

Numerous studies have demonstrated the biocontrol effects of *Bacillus* spp. on tomato pathogens due to the production of antibiotics (Table 2). Bouchard-Rochette et al. [90] reported a strong antagonistic effect of *B. pumilus* PTB180 and *B. subtilis* PTB185 against several plant pathogens, including *B. cinerea*, *F. oxysporum*, *R. solani*, *S. sclerotiorum*, *Pythium ultimum*, and *Phytophthora capsici*, due to the production of surfactin (both strains) as well as iturin and fengycin (*B. subtilis* PTB185). Moreover, foliar application of both strains individually and in a mixture significantly reduced the incidence of grey mould on tomatoes in greenhouse conditions. Strain *B. velezensis* NKMV-3, along with its lipopeptide extract, consisting of surfactin, iturin, and fengycin, effectively controlled *A. solani* on tomatoes in greenhouse studies [91]. Jia et al. [92] found that *B. amyloliquefaciens* XJ-BV2007 produces fengycin, which has an important role in the control of black spot disease of tomato and mycotoxins caused by *A. alternata*. Similarly, control of Fusarium wilt of tomato by *B. amyloliquefaciens* strain PPL was mainly due to the production of fengycins [93]. Two antagonistic strains, *B. subtilis* MB14 and *B. amyloliquefaciens* MB101, that showed a significant reduction of root rot symptoms in tomato caused by *R. solani*, were found positive for genes encoding surfactin, fengycin, bacillomycin, and iturin production [94]. PCR amplification revealed the presence of surfactin, fengycin, iturin, and bacilysin biosynthetic genes in the *B. amyloliquefaciens* Oj-2.16 that exhibited a high biocontrol efficacy against Verticillium wilt in tomato seedlings [95]. 

Additionally, *Bacillus* spp. are known to produce other non-ribosomally synthesised LPs (e.g., bacitracins, kustakins, polymixins), peptides (e.g., mycobacillin, bacilysin), and polyketides (e.g., difficidin, microlactin, bacillaene) with a wide array of antibacterial and antifungal activities [13,81]. For instance, Im et al. [96] isolated difficidin and oxydifficidin from the B. *methylotrophicus* DR-08 strain, which exert antagonistic effects against various pathogenic bacteria, including *R. solanacearum*, a causative agent of bacterial wilt in tomatoes. Furthermore, antimicrobial compounds macrolactin and bacillomycin D, with significant activity against *R. solanacearum* and *Fusarium oxysporum*, respectively, were isolated from biocontrol agent *B. amyloliquefaciens* NJN-6 [97]. *Bacillus amyloliquefaciens* DSBA-11 showed the highest inhibition of *Ralstonia pseudosolanacearum* compared to other *Bacillus* spp. due to the synthesis of polyketide antibiotics, viz., difficidin, macrolactin, and bacillaene [98]. Biosynthesis genes for macrolactin H, bacillaene, fengycin, difficidin, bactin, bacilysin, and surfactin were found in the strain *B. velezensis* SDTB038, explaining its biocontrol effects against Fusarium crown and root rot of tomato [99].

### 4.2. Lytic Enzymes

Synthesis of hydrolytic enzymes is an important mechanism employed by *Bacillus* spp. to suppress the target pathogens, in particular pathogenic fungi (Figure 8). A fibrous structure of the fungal cell wall predominately consists of polysaccharides, such as chitin, glucans, and mannans, as well as glycoproteins [100]. Lytic enzymes, like chitinases, chitosanases, glucanases, proteases, and cellulases, degrade the glycosidic bonds of such fungal cell wall structural components [101]. In addition to plant defence, hydrolytic enzymes also participate in plant growth and development [102].

Lytic enzymes produced by *Bacillus* spp. have been reported to suppress several tomato diseases (Table 2). For instance, *B. pumilus* SG2 produced two chitinases with hydrolytic activities on both oligosaccharide and polymeric substrates and an inhibitory effect on *Rhizoctonia solani*, *Stemphyllium botryosum*, *Verticillium* sp., *Bipolaris* sp., and *Nigrospora* sp. [103]. Fruit treatment with chitosanase-producing *B. subtilis* V26 significantly reduced postharvest decay of tomato caused by *B. cinerea* [104]. Additionally, *B. velezensis* KS04AU exhibited in vitro antagonism against *F. oxysporum*, *F. graminearum*, *A. alternata*, and *P. syringae*, as well as in vivo biocontrol against *F. oxysporum* f. sp. *radicis-lycopersici* due to chitinase, cellulase, amylase, protease, lipase, and phytase activities [105]. Several *Bacillus* spp. strains controlled Fusarium wilt in tomatoes caused by *F. oxysporum* f. sp. *lycopersici* due to superior cellulolytic and proteolytic activity [106].

### 4.3. Competition for Nutrients and Space

Competition for nutrients and space is a key physical mechanism that BCAs use to prevent the growth and spread of pathogens (Figure 8). It means that BCAs and plant pathogens occupy the same niches and have a simultaneous demand for the same resources (e.g., space; nutrients such as carbon, hydrogen, oxygen, phosphorus, nitrogen, and others) [107]. Competition for microelements, such as iron, manganese, copper, and zinc, also occurs between antagonists and pathogenic microorganisms in the soil [108]. *Bacillus* spp. are very efficient in solubilising and absorbing nutrients, thereby depleting resources and making the environment less favourable for the development of pathogens [88]. Furthermore, as biocontrol agents, *Bacillus* spp. have a good ability to colonise, survive, adapt, and tolerate different stress conditions, which facilitates their establishment and maintenance in the intended environment [109].

Recently, several studies have reported the inhibition of tomato pathogens by *Bacillus* spp. biocontrol agents due to competition for nutrients and space (Table 2). For instance, *B. velezensis* strain GF267 showed the highest reduction of tomato bacterial spot (pathogen *X. perforans*) and better competition ability than pathogens, as proven by the utilisation profile of carbon sources [110]. Tan et al. [111] revealed the growth promotion potential of *B. amyloliquefaciens* strains CM-2 and T-5, as well as their biocontrol effect against *R. solanacearum*, i.e., bacterial wilt, in greenhouse conditions, followed by high colonisation of both antagonists and decreased density of pathogens in the tomato rhizosphere. Similarly, *B. amyloliquefaciens* SQYUV 162 efficiently controlled *R. solanacearum* due to competition for root exudates between antagonists and pathogens [112].

### 4.4. Siderophores

Iron (Fe) is an essential micronutrient for numerous metabolic and signalling processes, including electron transport, photosynthesis, respiration, nitrogen fixation, and DNA synthesis. Iron availability in the soil is a limiting factor for both plants and microorganisms, since Fe is often present in its insoluble form, i.e., ferric oxide and hydroxide complexes. *Bacillus* spp. have evolved a mechanism for iron acquisition through the production of small metal-chelating protein compounds with a high affinity for ferric iron (Fe^3+^), known as siderophores (Figure 8) [113]. These bacteria produce a wide array of siderophores, such as bacillibactin, pyochelin, pyoverdine, and petrobactin [13]. Siderophores play an important role in biological control, making Fe unavailable to soil-borne pathogens [76]. Siderophores produced by *Bacillus* spp. and other biocontrol agents have a much higher affinity for iron than the siderophores produced by plant pathogens [114]. Additionally, siderophores have the ability to bind a wide range of other metals and act as bioremediation and plant growth-promoting agents [115].

Kalam et al. [116] reported that all selected *Bacillus* spp. isolates from the tomato rhizosphere produced siderophores, along with other plant growth-promoting and antagonistic traits. Siderophore-producing *Bacillus* strains have been directly involved in the subsequent inhibition of different tomato pathogens (Table 2). Xu et al. [117] recorded a reduction of tomato grey mould and growth promotion of tomato seedlings in a greenhouse using *B. amyloliquefaciens* SG08-09 and *B. subtilis* SG09-12 that produced siderophores, protease, cellulase, and IAA. Similarly, the *B. velezensis* RC116 strain demonstrated protease, lipase, and amylase activities; produced siderophores and IAA; and showed strong antimicrobial activity towards *R. solanacearum* and *F. oxysporum* f. sp. *lycopersici*, as well as biocontrol effects against bacterial wilt in a greenhouse setting [118]. *B. amyloliquefaciens* strain S1 showed the production of siderophores as well as chitinase, cellulase, protease, lipase, and antagonistic activities against bacterial canker (pathogen *C. michiganensis* ssp. *michiganensis*) of tomato in net house conditions [119].

### 4.5. Volatile Compounds

*Bacillus* biocontrol agents can produce numerous volatile secondary metabolites with a broad spectrum of antimicrobial activity (Figure 8). Volatiles produced by *Bacillus* spp. involve different organic (alcohols, alkenes, benzenoids, ketones, pyrazines, terpenes) and inorganic (e.g., NH_3_, HCN, H_2_S, NO_2_, CO_2_) compounds [120]. Such compounds have a crucial role in improving plant response and tolerance to various biotic and abiotic stresses. Volatile compounds also promote plant growth and development and improve water and nutrient acquisition [121]. The most volatile compounds come from glucose oxidation, fermentations, carbon metabolism, amino acid degradation, and sulphate reduction [122]. It has been demonstrated that volatile compounds from *Bacillus* spp. effectively inhibited the growth of tomato pathogens (Table 2). Thus, *B. subtilis* EPCO16 suppressed the growth of *F. oxysporum* f. sp. *lycopersici* and promoted the growth of tomato seedlings due to the production of siderophore, HCN, chitinase, β-1,3-glucanase, and protease [123]. Native bacterial isolates *B. subtilis* BS6 and *B. subtilis* CS13 significantly reduced the growth of tomato pathogens *A. solani* and *F. oxysporum* f. sp. *lycopersici* under in vitro conditions due to the production of NH_3_ as well as chitinase, cellulase, and protease activities [124]. An endophytic *B. subtilis* EB-28 strain, positive for H_2_S production, showed strong antifungal activity against the tomato pathogen *B. cinerea*, with the growth inhibition of 71% in vitro and 52% in vivo [125]. The consortium of volatile organic compounds (benzenes, ketones, aldehydes, alkanes, acids, furan, and naphthalene) produced by *B. amyloliquefaciens* T-5 showed a very strong antagonistic effect on the virulence and growth of the tomato pathogen *R. solanacearum* [126]. *B. subtilis* BS-01 significantly reduced early blight disease severity (pathogen *A. solani*) on tomato foliage due to the production of volatile organic compounds (triphenylphosphine oxide, *n*-hexadecanoic acid, octadecanoic acid, octadecane, eicosane, dodecyl acrylate, and others) [127]. Guo et al. [128] reported a strong antifungal activity of *Bacillus tequilensis* XK29 volatile compounds against the *B. cinerea* (postharvest decay) of cherry tomatoes both under in vitro and in vivo conditions.

**Table 2:** *Bacillus* spp. biocontrol agents applied in the control of tomato diseases and their mechanisms of action.

Bacillus Strain	Part of Tomato	Pathogen/Disease	Mode of Action	Reference
*B. pumilus* PTB180 *B. subtilis* PTB185	Leaves	*B. cinerea*/grey mould	Surfactin/surfactin, iturin, fengycin	[90]
*B. velezensis* NKMV-3	Leaves	*A. solani*/early blight	Surfactin, iturin, fengycin	[91]
*B. amyloliquefaciens* XJ-BV2007	Fruits	*A. alternata*/black spot	Fengycin	[92]
*B. amyloliquefaciens* PPL	Plants	*Fusarium oxysporum* f. sp. *lycopersici*/Fusarium wilt	Fengycin	[93]
*B. subtilis* MB14 *B. amyloliquefaciens* MB101	Roots	*R. solani/*root rot	Surfactin, fengycin bacillomycin, iturin	[94]
*B. amyloliquefaciens* Oj-2.16	Plants	*V. dahliae*/verticillium wilt	Surfactin, iturin, fengycin, bacilysin	[95]
B. *methylotrophicus* DR-08	Plants	*R. solanacearum*/bacterial wilt	Difficidin, oxydifficidin	[96]
*B. amyloliquefaciens* DSBA-11	Plants	*R. solanacearum*/bacterial wilt	Difficidin, macrolactin, bacillaene	[98]
*B. velezensis* SDTB038	Plants	*Fusarium oxysporum* f. sp. *radicis-lycopersici*/Fusarium crown and root rot wilt	Macrolactin H, bacillaene, fengycin, difficidin, bactin, bacilysin, surfactin	[99]
*B. subtilis* V26	Fruits	*B. cinerea*/grey mould	Chitosanase	[104]
*B. velezensis* KS04AU	Roots	*F. oxysporum* f. sp. *radicis-lycopersici*/Fusarium rot	Chitinase, cellulase, amylase, protease, lipase, phytase	[105]
Several *Bacillus* spp.	Plants	*Fusarium oxysporum* f. sp. *lycopersici*/Fusarium wilt	Cellulase, protease	[106]
*B. velezensis* GF267	Plants	*X. perforans*/bacterial spot	Competition for nutrients and space	[110]
*B. amyloliquefaciens* CM-2 and T-5	Plants	*R. solanacearum*/bacterial wilt	Competition for nutrients and space	[111]
*B. amyloliquefaciens* SQYUV 162	Plants	*R. solanacearum*/bacterial wilt	Competition for nutrients and space	[112]
*B. amyloliquefaciens* SG08-09 *B. subtilis* SG09-12	Plants	*B. cinerea*/grey mould	Siderophores, protease, cellulase, ammonia, IAA	[117]
*B. velezensis* RC116	Plants	*R. solanacearum*/bacterial wilt	Protease, amylase, lipase, siderophores, IAA	[118]
*B. amyloliquefaciens* S1	Plants	*C. michiganensis* ssp. *michiganensis*/bacterial canker	Siderophores, chitinase, cellulase, protease, lipase	[119]
*B. subtilis* EB-28	Leaves	*B. cinerea*/grey mould	Hydrogen sulphide	[125]
*B. subtilis* BS-01	Leaves	*A. solani*/early blight	Volatile organic compounds	[127]
*B. tequilensis* XK29	Fruits	*B. cinerea*/grey mould	Volatile compounds	[128]
*B. subtilis* BS 21-1	Plants	*B. cinerea*/Botrytis rot	Induced resistance	[129]
*B. aryabhattai* SRB02	Plants	*F. oxysporum* f. sp. *lycopersici*/Fusarium wilt	Induced resistance	[15]
*B. subtilis* OTPB1	Leaves	*P. infestans*/late blight *A. solani*/early blight	Induced resistance	[130]
*B. amyloliquefaciens FZB42*	Stems	*S. sclerotiorum*/Sclerotinia rot	Induced resistance	[131]
*B. cabrialesii BH5*	Leaves	*B. cinerea*/grey mould	Induced resistance	[132]
*B. velezensis* YYC	Leaves	*P. solanacearum*/bacterial wilt	Induced resistance	[133]
*B. subtilis* CBR05	Leaves	*X. campestris* pv. *vesicatoria*/ bacterial spot	Induced resistance	[134]
*B. subtilis* SR22	Roots	*R. solani*/Rhizoctonia rot	Induced resistance	[135]

### 4.6. Induced Resistance

Plants exposed to biotic stress have adapted by developing various defence responses, including induced systemic resistance (ISR) (Figure 8). Induced resistance is elicited by beneficial microorganisms, such as biocontrol agents, before infection [136]. Multiple strains of *Bacillus* spp. have been reported to stimulate plant defence responses in tomato plants (Table 2). *B. subtilis* BS 21-1 could be used as a plant growth-promoting and biocontrol agent for the control of Botrytis rot disease in tomatoes through systemic resistance [129]. ISR is mainly dependent on the jasmonic acid/ethylene (JA/ET) signalling. Recently, the salicylic acid (SA) pathway was also proven to be involved in plant recognition of biocontrol agents. For instance, *Bacillus aryabhattai* SRB02 significantly inhibited tomato wilt disease caused by *F. oxysporum* f. sp. *lycopersici* and promoted plant growth by modulating endogenous hormones (SA, JA) [15]. Dimopoulou et al. [137] revealed that tomato defence signalling pathways depended on the dose of application. Thus, a lower dose of a commercial bacterial product based on biocontrol agent *B*. *amyloliquefaciens* MBI600 activated SA-responsive genes; a higher dose primed defence via JA/ET signalling; and the suggested dose induced synergistic cross-talk between both pathways. 

Additionally, ISR is associated with the accumulation of defence-related enzymes, including peroxidase (POX), polyphenol oxidase (PPO), phenylalanine ammonia-lyase (PAL), superoxide dismutase (SOD), and catalase (CAT) [138]. *Bacillus* spp. may trigger ISR in plants through the action of plant hormones, antibiotics, volatiles, and other bioactive metabolites. For instance, Ongena et al. [139] reported that lipopeptide compounds such as surfactins and fengycins, produced by *Bacillus* spp., may also be involved in the elicitation of ISR. Simiraly, *B. subtilis* OTPB1 increased plant growth and seedling vigour index, exhibited in vitro antifungal activity towards *P. infestans* and *A. solani*, and enhanced systemic resistance in tomato seedlings against late and early blight via the induction of plant hormones (IAA, GA_3_) and defence enzymes (POX, PPO, and SOD) [130]. The biocontrol agent *B. amyloliquefaciens* FZB42 inhibited the growth of *S. sclerotiorum* and reduced lesion size in tomato plants under in vitro and greenhouse conditions, respectively. Moreover, it was found that the antifungal activity of *B. amyloliquefaciens* FZB42 was a result of lipopeptide fengycin, which induced systemic resistance in tomato and downregulated the expression of defence-related genes in tomato plants [131]. Zhou et al. [132] demonstrated that both *Bacillus cabrialesii* BH5 and fengycin H, produced by BH5, stimulated the ISR of tomato plants against *B. cinerea* through JA signalling and had a significant biocontrol effect under in vivo conditions. Moreover, *B. velezensis* YYC significantly reduced bacterial wilt caused by *Pseudomonas solanacearum* in tomato plants in vivo and enhanced plant resistance by increasing the activity of defence-related enzymes (PAL, POD, and SOD) while inducing the expression of genes related to IAA, GA, JA, and SA [133]. Chandrasekaran et al. [134] suggested that the β-1,3-glucanase and phenylalanine ammonia-lyase activities of *B. subtilis* CBR05 are responsible for tomato resistance against bacterial spot disease caused by *X. campestris* pv. *vesicatoria*. Biocontrol agent *B. subtilis* SR22 efficiently suppressed the *R. solani* growth and root rot disease under in vitro and greenhouse conditions due to the production of numerous bioactive compounds, including phthalic acid, pyrrolo[1,2-a]pyrazine-1,4-dione, hexahydro, chlorogenic acid, propyl thioglycolic acid, and 2,3-butanediol [135]. Moreover, this strain improved tomato growth parameters as well as total phenolic content and antioxidant enzyme activity in tomato roots, indicating its ISR effect [135]. The application of *Bacillus subtilis* MBI600 on tomato plants significantly improved plant growth and had a strong biocontrol effect against three tomato pathogens, namely, *R. solani*, *Pythium ultimum*, and *F. oxysporum* f. sp. *radicis-lycopersici* [140]. Furthermore, activation of two auxin- and defence-related genes used as markers of the SA and JA/ET signalling pathways suggested that the strain MBI600 induced systemic resistance in tomato plants [140].

## 5. Combined Strategies for Tomato Disease Management

*Bacillus* spp. still cannot completely replace the use of agrochemicals due to their limited efficacy in natural conditions. Therefore, further research is needed to provide more effective control of tomato diseases. In tomato production, different strategies have been combined to achieve integrated and more effective control of plant pathogens. Using a particular strategy individually does not meet the needs for reducing tomato yield losses while suppressing plant diseases and maintaining agricultural sustainability. Nowadays, integrated disease management (IDM) of vegetable crops, including tomato, is a common approach in modern agriculture and implies using all available measures, including host plant resistance and cultural, biological, and chemical control, that ensure high yield and quality in accordance with economic, social, and ecological principles [61].

Biocontrol agents are an important component of an IDM that can significantly minimise the need for agrochemicals and the presence of chemical residues in agricultural products. One of the most common approaches in the suppression of tomato pathogens is the combined application of *Bacillus* spp. with chemical fertilisers or PPPs (Table 3). For example, foliar application of B. subtilis alone or in combination with plant nutrients (NPK, Zn, Mg, and B) significantly reduced *A. solani* by 67–83%; improved the growth of tomato plants by 20–77%; and modified the content of total chlorophyll, carotenoids, phenols, and antioxidant enzymes [141]. These results showed a synergistic effect of biocontrol agents and plant nutrients for protection against early blight disease and the improvement of the growth of tomato plants. Moreover, the combined application of NPK fertiliser and two biocontrol agents, *B. subtilis* and *T. asperellum*, in the management of damping-off disease caused by Pythium aphanidermatum resulted in a significantly higher dry mass of tomato seedlings as compared to fertiliser or either biocontrol agent alone [142].

Furthermore, the use of *Bacillus* spp. with organic fertiliser is also more effective in controlling tomato diseases than the individual application of biocontrol agents (Table 3). Thus, *B. amyloliquefaciens*, SQY 162 applied with cattle manure compost and amino acid fertiliser was efficient in suppressing tomato bacterial wilt caused by R. solanacearum [143]. Similarly, the most effective protection of tomatoes from *P. infestans* and the highest effect on tomato growth were achieved with *B. subtilis* subsp. *subtilis* and oak bark compost, suggesting their mutual contribution to soil quality and plant resistance to late blight [144]. Ji et al. [145] showed full compatibility of *B. methylotrophicus* TA-1 with the fungicide fluopimomide and their synergistic effect against the grey mould of tomato in laboratory, greenhouse, and field trials, indicating the possibility of reducing the amounts of fungicide application. Also, the growth of *B. subtilis* B-001 was unaffected by the Saisentong in vitro, while their combination resulted in the higher control of *R. solanacearum*, a causative agent of tomato bacterial wilt, compared with either bacterial or bactericide treatment in both greenhouse and field conditions [146]. Bacilli strains, i.e., *B. subtilis* GB03 and FZB24, *B. amyloliquefaciens* IN937a, and *B. pumilus* SE34, combined with acibenzolar-*S*-methyl and hymexazol, significantly increased the suppression of Fusarium crown and root rot of tomato caused by *F. oxysporum* f. sp. *radicis-lycopersici* [147]. A base soil treatment of *Bacillus* spp. or *B. subtilis* combined with foliar-applied *Reynoutria sachalinensis*, *Melaleuca alternifolia*, harpin αβ proteins, or bee honey efficiently reduced the intensity of grey mould (*B. cinerea)* and powdery mildew (*Leveillula taurica)* as compared to conventional foliar disease control [148]. Mousa et al. [149] showed that *B. amyloliquefaciens* BA, alone or in combination with peppermint oil, promoted seed germination and seedling vigour and reduced the severity of Fusarium wilt in tomato (pathogen *F. oxysporum* f. sp. *lycopersici*) under greenhouse and field conditions.

Another promising strategy for controlling tomato diseases is the use of consortia-based biocontrol agents (Table 3). For instance, Abdeljalil et al. [150] reported that the combination of three biocontrol bacterial agents, *B. subtilis*, *B. thuringiensis*, and *Enterobacter cloacae*, with or without biocontrol oomycete *Pythium oligandrum*, significantly reduced the occurrence of root rot caused by *R. solani*, with microbial treatments being more effective than fungicide. Aside from their antifungal potential, *B. thuringiensis* strains are primarily applied as biopesticides for intentional insect control, while an effective washing procedure could reduce the bacterial residues on tomato fruits for safe food consumption [151]. Similarly, Chien and Huang [152] reported that the single or combined use of two bioagents, *B. amyloliquefaciens* and *T. asperellum*, has the potential to control tomato bacterial spot caused by *X. perforans*, producing statistically equal or better results compared to copper standard fungicide (cupric hydroxide with ethylene bisdithiocarbamate). Microbial antagonists, *B. subtilis*, *P. fluorescens*, and *Trichoderma* spp., significantly reduced the early blight disease in greenhouse and field conditions, while their biocontrol potential was comparable with the fungicide effect [153]. Application of *B. velezensis* ERBS51 and *Bacillus* sp. ERBS10 with arbuscular mycorrhiza fungi (*Funneliformis mosseae* and *Glomus fasciculatum*) had the highest effect on the suppression of Fusarium wilt as well as tomato growth and yield in pot and field experiments [154]. Furthermore, two strains with plant growth-promoting and biocontrol potential, *B. subtilis* PPB9 and *Stenotrophomonas maltophilia* PPB3, increased seed germination, seedling vigour, plant growth, chlorophyll content, and nutrient concentration (N, P, K) and reduced Southern blight disease of tomato in greenhouse and field conditions [155]. The utilisation of different biocontrol agents caused the desired microbiome shifts, which contribute to plant protection against the target pathogen. Thus, Elsayed et al. [156] showed that *B. velezensis* B63 and *P. fluorescens* P142 significantly reduced bacterial wilt caused by *R. solanacearum* B3B, accompanied by lower pathogen abundance and shifts in the prokaryotic community composition of the tomato rhizosphere. Similarly, antagonistic bacteria *B. velezensis* MB101 and *Pseudomonas fluorescens* MPF47 significantly influenced the bacterial count and function, as well as soil enzymes, with the beneficial effect of examined carbon sources on healthy microbiome propagation towards *R. solani* in the tomato rhizosphere [157]. Additionally, Khalil [158] recorded that *B. subtilis*, *Trichoderma viride*, and Topsin-M70 significantly suppressed Fusarium root rot in tomatoes, although the fungicide was the most efficient treatment. However, microbial antagonists had a positive influence on the rhizosphere microbiome and enzyme activity as compared to fungicide.

**Table 3:** Combined application of *Bacillus* spp. and other methods/agents in the control of tomato diseases.

Bacillus Strain	Combination with Antagonists/Fungicide	Pathogen/Disease	Reference
*B. subtilis*	Plant nutrients (NPK, Zn, Mg, B)	*A. solani*/early blight	[141]
*B. subtilis*	NPK fertiliser *Trichoderma asperellum*	*Pythium aphanidermatum*/damping off	[142]
*B. amyloliquefaciens*	Cattle manure compost Amino acid fertiliser	*R. solanacearum*/bacterial wilt	[143]
*B. subtilis* subsp. *subtilis*	Oak-bark compost	*P. infestans*/late blight	[144]
*B. methylotrophicus*	Fungicide fluopimomide	*B. cinerea*/grey mould	[145]
*B. subtilis* B-001	Bactericide Saisentong	*R. solanacearum*/bacterial wilt	[146]
*B. subtilis* *B. amyloliquefaciens* *B. pumilus*	Acibenzolar-S-methyl hymexazol	*F. oxysporum* f. sp. *radicis-lycopersici*/Fusarium crown and root rot	[147]
*Bacillus* spp. *B. subtilis*	*Reynoutria sachalinensis* *Malaleuca alternifolia* Harpin αβ proteins Bee honey	Grey mould/*B. cinerea* Powdery mildew/*Leveillula taurica*	[148]
*B. amyloliquefaciens*	Peppermint oil	*F. oxysporum* f. sp. *lycopersici*/Fusarium wilt	[149]
*B. subtilis* *B. thuringiensis*	*Enterobacter cloacae* *Pythium oligandrum*	*R.solani*/Rhizoctonia root rot	[150]
*B. amyloliquefaciens*	*Trichoderma asperellum*	*X. perforans*/bacterial spot	[152]
*B. subtilis*	*Trichoderma* spp. *Pseudomonas fluorescens*	*A.solani*/early blight	[153]
*Bacillus* sp. *B. velezensis*	*Funneliformis mosseae* *Glomus fasciculatum*	*Fusarium oxysporum* f. sp. *lycopersici*/Fusarium wilt	[154]
*B. subtilis* PPB9	*Stenotrophomonas maltophila* PPB3	*Sclerotium rolfsii*/Southern blight	[155]
*B. velezensis*	*P. fluorescens*	*R. solanacearum*/bacterial wilt	[156]
*B. velezensis*	*P. fluorescens*	*R.solani*/Rhizoctonia root rot	[157]
*B. subtilis*	*Trichoderma viride*	*Fusarium solani*/Fusarium root rot	[158]

## 6. Summary and Conclusions

Tomato production can be threatened by various phytopathogenic fungi and bacteria that affect yield reduction and fruit quality. Tomato protection from plant pathogens still heavily relies on the application of PPPs. In order to provide high-quality food, the imperative for the protection of tomatoes is the introduction of alternative pathogen control measures. One of the most promising strategies for reducing the use of PPPs and suppressing pathogens is the use of *Bacillus* spp. and *Bacillus*-based PPPs. However, the wider practical application of these agents is frequently confronted with limited and unstable efficiency in field conditions. Dynamic and complex soil–plant–microbe interactions, accompanied by biotic and abiotic stress and the effects of climate change, influence the colonisation and action of introduced *Bacillus* spp. agents. Nowadays, new approaches have been proposed to improve biocontrol efficacy, including the combined application of *Bacillus* spp. with organic or chemical amendments, as well as the use of antimicrobial metabolites with or without biocontrol agents. Furthermore, using a microbial consortium consisting of *Bacillus* spp. strains and other biocontrol or plant growth-promoting agents with multiple functions showed higher survival, adaptability, and effectiveness as compared with their individual applications. Integrated multi-omics and bioinformatics technologies should be exploited to underline the mechanisms and efficiency issues of *Bacillus* spp. agents for managing plant diseases in sustainable agricultural production.

## References

[1] FAOSTAT Database Food and Agriculture Organization Statistics. https://www.fao.org/faostat/en/.

[2] Ali M.-Y., Sina A.A.I., Khandker S.S., Neesa L., Tanvir E.M., Kabir A., Khalili M.I., Gan S.H. (2018). Nutritional composition and bioactive compounds in tomatoes and their impact on human health and disease: A review. Foods.

[3] Panno S., Davino S., Caruso A.G., Bertacca S., Crnogorac A., Mandić A., Noris E., Matić S. (2021). A Review of the most common and economically important diseases that undermine the cultivation of tomato crop in the Mediterranean Basin. Agronomy.

[4] Montenegro I., Madrid A., Cuellar M., Seeger M., Alfaro J.F., Besoain X., Martínez J.P., Ramirez I., Olguín Y., Valenzuela M. (2018). Biopesticide activity from drimanic compounds to control tomato pathogens. Molecules.

[5] Attia M.S., El-Wakil D.A., Hashem A.H., Abdelaziz A. (2022). Antagonistic effect of plant growth-promoting fungi against *fusarium* wilt disease in tomato: In vitro and in vivo study. Appl. Biochem. Biotechnol..

[6] Wang Y., Zhang Y., Gao Z., Yang W. (2018). Breeding for resistance to tomato bacterial diseases in China: Challenges and prospects. Hortic. Plant J..

[7] Butsenko L., Pasichnyk L., Kolomiiets Y., Kalinichenko A. (2020). The effect of pesticides on the tomato bacterial speck disease pathogen *Pseudomonas syringae* pv. tomato. Appl. Sci..

[8] Ma M., Taylor P.W.J., Chen D., Vaghefi N., He J.Z. (2023). Major soilborne pathogens of field processing tomatoes and management strategies. Microorganisms.

[9] Tudi M., Ruan H.D., Wang L., Lyu J., Sadler R., Connell D., Chu C., Phung D.T. (2021). Agriculture development, pesticide application and its impact on the environment. Int. J. Environ. Res. Public Health.

[10] Tripathi S., Srivastava P., Devi R.S., Bhadouria R. (2020). Influence of synthetic fertilizers and pesticides on soil health and soil microbiology. Agrochemicals Detection, Treatment and Remediation.

[11] He D.C., He M.H., Amalin D.M., Liu W., Alvindia D.G., Zhan J. (2021). Biological control of plant diseases: An evolutionary and eco-economic consideration. Pathogens.

[12] Jacobsen B.J., Zidack N.K., Larson B.J. (2004). The role of *Bacillus*-based biological control agents in integrated pest management systems: Plant diseases. Phytopathology.

[13] Miljaković D., Marinković J., Balešević-Tubić S. (2020). The significance of *Bacillus* spp. in disease suppression and growth promotion of field and vegetable crops. Microorganisms.

[14] Taha N.A., Elsharkawy M.M., Shoughy A.A., El-Kazzaz M.K., Khedr A.A. (2023). Biological control of postharvest tomato fruit rots using *Bacillus* spp. and *Pseudomonas* spp.. Egypt. J. Biol. Pest Control.

[15] Syed Nabi R.B., Shahzad R., Tayade R., Shahid M., Hussain A., Ali M.W., Yun B.W. (2021). Evaluation potential of PGPR to protect tomato against *Fusarium* wilt and promote plant growth. PeerJ.

[16] Zhang B., Zhang Y., Liang F., Ma Y., Wu X. (2019). An extract produced by *Bacillus* sp. BR3 influences the function of the GacS/GacA two-component system in *Pseudomonas syringae* pv. *tomato* DC3000. Front. Microbiol..

[17] Jones J.B., Zitter T.A., Momol M.T., Miller S.A. (2016). Compendium of Tomato Diseases and Pests.

[18] Ahmed F.A., Sipes B.S., Alvares A.M. (2017). Postharvest diseases of tomato and natural products for disease management. Afr. J. Agric. Res..

[19] Rodrigues M.H.P., Furlong E.B. (2022). Fungal diseases and natural defense mechanisms of tomatoes (*Solanum lycopersicum*): A review. Physiol. Mol. Plant Pathol..

[20] Kumar S., Singh R., Kashyap P.L., Srivastava A.K. (2013). Rapid detection and quantification of *Alternaria solani* in tomato. Sci. Hortic..

[21] Mugao L. (2023). Morphological and molecular variability of *Alternaria solani* and *Phytophthora infestans* causing tomato blights. Int. J. Microbiol..

[22] Chaerani R., Voorrips R. (2006). Tomato early blight (*Alternaria solani*): The pathogen, genetics, and breeding for resistance. J. Gen. Plant Pathol..

[23] Ávila M.C.R., Lourenço V.L., Quezado-Duval A.M., Becker W.F., Abreu-Tarazi M.F., Borges L.C., Nascimento A.D.R. (2020). Field validation of TOMCAST modified to manage Septoria leaf spot on tomato in the central-west region of Brazil. Crop Prot..

[24] Silva B.N., Picanco B.B.M.P., Hawerroth C., Silva L.C., Rodrigues F.A. (2022). Physiological and biochemical insights into induced resistance on tomato against septoria leaf spot by a phosphite combined with free amino acids. Physiol. Mol. Plant Pathol..

[25] Mani S.D., Pandey S., Govindan M., Muthamilarasan M., Nagarathnam R. (2021). Transcriptome dynamics underlying elicitor-induced defense responses against Septoria leaf spot disease of tomato (*Solanum lycopersicum* L.). Physiol. Mol. Biol. Plants.

[26] Poveda J., Barquero M., González-Andrés F. (2020). Insight into the microbiological control strategies against *Botrytis cinerea* using systemic plant resistance activation. Agronomy.

[27] Li H., Chen Y., Zhang Z., Li B., Qin G., Tian S. (2018). Pathogenic mechanisms and control strategies of *Botrytis cinerea* causing post-harvest decay in fruits and vegetables. Food Qual. Saf..

[28] Fillinger S., Elad Y. (2016). Botrytis—The Fungus, the Pathogen and Its Management in Agricultural Systems.

[29] Srinivas C., Devi D.N., Murthy K.N., Mohan C.D., Lakshmeesha T.R., Singh B.P., Kalagatur N.K., Niranjana S.R., Hashem A., Alqarawi A.A. (2019). *Fusarium oxysporum* f. sp. *lycopersici* causal agent of vascular wilt disease of tomato: Biology to diversity—A review. Saudi J. Biol. Sci..

[30] Ponsankar A., Senthil-Nathan S., Vasantha-Srinivasan P., Pandiyan R., Karthi S., Kalaivani K., Chellappandian M., Narayanaswamy R., Thanugaivel A., Patcharin K. (2023). Systematic induced resistance in *Solanum lycopersicum* (L.) against vascular wilt pathogen (*Fusarium oxysporum* f. sp. *lycopersici*) by *Citrullus colocynthis* and *Trichoderma viride*. PLoS ONE.

[31] Pandey A.K., Dinesh K., Nirmala N.S., Kumar A., Chakraborti D., Bhattacharyya A. (2023). Insight into tomato plant immunity to necrotrophic fungi. Curr. Res. Biotechnol..

[32] Li X., Wang Q., Li H., Wang X., Zhang R., Yang X., Jiang Q., Shi Q. (2023). Revealing the mechanisms for linalool antifungal activity against *Fusarium oxysporum* and its efficient control of Fusarium wilt in tomato plants. Int. J. Mol. Sci..

[33] Aravena R., Besoain X., Riquelme N., Salinas A., Valenzuela M., Oyanedel E., Barros W., Olguin Y., Madrid A., Alvear M. (2021). Antifungal nanoformulation for biocontrol of tomato root and crown rot caused by *Fusarium oxysporum* f. sp. *radicis-lycopersici*. Antibiotics.

[34] Acharya B., Ingram T.W., Oh Y., Adhikari T.B., Dean R.A., Louws F.J. (2020). Opportunities and challenges in studies of host-pathogen interactions and management of *Verticillium dahliae* in tomatoes. Plants.

[35] Retief E., Lamprecht S., McLeod A. (2023). Characterisation and pathogenicity of *Verticillium dahliae* isolates associated with Verticillium wilt of tomato in the Limpopo Province of South Africa. J. Plant Pathol..

[36] Fradin E.F., Thomma B.P. (2006). Physiology and molecular aspects of Verticillium wilt diseases caused by *V. dahliae* and *V. albo-atrum*. Mol. Plant Pathol..

[37] Buhtz A., Hohe A., Schwarz D., Grosch R. (2017). Effects of *Verticillium dahliae* on tomato root morphology considering plant growth response and defence. Plant Pathol..

[38] Garcia P.G., Neves dos Santos F., Zanotta S., Eberlin M.N., Carazzone C. (2018). Metabolomics of *Solanum lycopersicum* Infected with *Phytophthora infestans* Leads to Early Detection of Late Blight in Asymptomatic Plants. Molecules.

[39] Mazumdar P., Singh P., Kethiravan D., Ramathani I., Ramakrishnan N. (2021). Late blight in tomato: Insights into the pathogenesis of the aggressive pathogen *Phytophthora infestans* and future research priorities. Planta.

[40] Maxim A., Albu V.C., Vodnar D.C., Mihăiescu T., Mang Ș.M., Camele I., Trotta V., Bonomo M.G., Mihalescu L., Sandor M. (2023). Assessment of Tomato (*Solanum lycopersicum*) Landraces for Their Agronomic, Biochemical Characteristics and Resistance to *Phytophthora infestans*. Agronomy.

[41] Skliros D., Papazoglou P., Gkizi D., Paraskevopoulou E., Katharios P., Goumas D.E., Tjamos S., Flemetakis E. (2023). *In planta* interactions of a novel bacteriophage against *Pseudomonas syringae* pv. *tomato*. Appl. Microbiol. Biotechnol..

[42] Basim H., Basim E., Yilmaz S., Dickstein E.R., Jones J.B. (2004). An outbreak of bacterial speck caused by *Pseudomonas syringae* pv. *tomato* on tomato transplants grown in commercial seedling companies located in the Western Mediterranean Region of Turkey. Plant Dis..

[43] Santamaría-Hernando S., López-Maroto Á., Galvez-Roldán C., Munar-Palmer M., Monteagudo-Cascales E., Rodríguez-Herva J.J., Tino Krell T., López-Solanilla E. (2022). Pseudomonas syringae pv. *tomato* infection of tomato plants is mediated by GABA and l-Pro chemoperception. Mol. Plant Pathol..

[44] Chai A., Ben H.Y., Guo W.T., Shi Y.X., Xie X.W., Li L., Li B.J. (2020). Quantification of viable cells of *Pseudomonas syringae* pv. *tomato* in tomato seed using propidium monoazide and a real-time PCR assay. Plant Dis..

[45] Yang P., Zhao L., Gao Y.G., Xia Y. (2023). Detection, Diagnosis, and Preventive Management of the Bacterial Plant Pathogen *Pseudomonas syringae*. Plants.

[46] Wang L., Tian Q., Zhou P., Zhao W., Sun X. (2022). Evaluation of droplet digital PCR for the detection of black canker disease in tomato. Plant Dis..

[47] Abo-Elyousr K.A.M., Bagy H.M.M.K., Hashem M., Alamri S.A.M., Mostafa Y.S. (2019). Biological control of the tomato wilt caused by *Clavibacter michiganensis* subsp. *michiganensis* using formulated plant growth-promoting bacteria. Egypt. J. Biol. Pest Control.

[48] Tripathi R., Vishunavat K., Tewari R. (2022). Morphological and molecular characterization of *Clavibacter michiganensis* subsp. *michiganensis* causing bacterial canker in tomatoes. Physiol. Mol. Plant Pathol..

[49] Malliarakis D., Pagoulatou M.G., Mpalantinaki E., Trantas E., Ververidis F., Goumas D.E. (2023). Phylogenetic diversity of *Clavibacter michiganensis* subsp. *michiganensis* isolates causing bacterial canker of tomato in Greece. J. Plant Pathol..

[50] Abrahamian P., Klein-Gordon J.M., Jones J.B., Vallad G.E. (2021). Epidemiology, diversity, and management of bacterial spot of tomato caused by *Xanthomonas perforans*. Appl. Microbiol. Biotechnol..

[51] Baldwin T.K., Woudt B., Lastdrager J., Berendsen S., Koenraadt H. (2023). Development and validation of real-time PCR tests for specific identification of *Xanthomonas* species causing bacterial spot disease on tomato (*Solanum lycopersicum*) and pepper (*Capsicum annuum*). EPPO Bull..

[52] Bernal E., Rotondo F., Roman-Reyna V., Klass T., Timilsina S., Minsavage G.V., Iruegas-Bocardo F., Goss E.M., Jones J.B., Jacobs J.M. (2022). Migration Drives the Replacement of *Xanthomonas perforans* races in the absence of widely deployed resistance. Front. Microbiol..

[53] Bamazi B., Banito A., Ayisah K.D., Sikirou R., Paret M.L., Kunwar S., Kamde K., Tchalla P., Afole S.L.N.A. (2022). Distribution and incidence of tomato bacterial wilt caused by *Ralstonia solanacearum* in the Central Region of Togo. Plant Health Prog..

[54] Mekonnen H., Kibret M., Assefa F. (2022). Plant growth promoting rhizobacteria for biocontrol of tomato bacterial wilt caused by *Ralstonia solanacearum*. Int. J. Agron..

[55] Rivera-Zuluaga K., Hiles R., Barua P., Caldwell D., Iyer-Pascuzzi A.S. (2023). Getting to the root of *Ralstonia* invasion. Sem. Cell Dev. Biol..

[56] Caldwell D., Kim B.S., Iyer-Pascuzzi A.S. (2017). *Ralstonia solanacearum* differentially colonizes roots of resistant and susceptible tomato plants. Phytopathology.

[57] Kruidhof H.M., Elmer W.H., Gullino M., Albajes R., Nicot P. (2020). Cultural methods for greenhouse pest and disease management. Integrated Pest and Disease Management in Greenhouse Crops.

[58] Baysal-Gurel F., Gardener B.M., Miller S.A. (2012). Soil Borne Disease Management in Organic Vegetable Production. Org. Agric..

[59] Lee Y., Choi C., Kim S.H., Yun J., Chang S., Kim Y.S., Hong J. (2012). Chemical pesticides and plant essential oils for disease control of tomato bacterial wilt. Plant Pathol. J..

[60] Lima G.P.P., Gómez H.A.G., Seabra Junior S., Maraschin M., Tecchio M.A., Borges C.V. (2022). Functional and nutraceutical compounds of tomatoes as affected by agronomic practices, postharvest management, and processing methods: A mini review. Front. Nutr..

[61] Kumari D., Gopireddy B.M., Kumari A., Vijaya M., Suresh V., Nayak H. (2020). Integrated disease management in tomato. J. Entomol. Zool. Stud..

[62] Ayaz M., Li C.-H., Ali Q., Zhao W., Chi Y.-K., Shafiq M., Ali F., Yu X.-Y., Yu Q., Zhao J.-T. (2023). Bacterial and fungal biocontrol agents for plant disease protection: Journey from lab to field, current status, challenges, and global perspectives. Molecules.

[63] O’Brien P.A. (2017). Biological control of plant diseases. Australas. Plant Pathol..

[64] Stenberg J.A., Sundh I., Becher P.G., Björkman C., Dubey M., Egan P.A., Friberg H., Gil J.F., Jensen D.F., Jonsson M. (2021). When is it biological control? A framework of definitions, mechanisms, and classifications. J. Pest Sci..

[65] Heydari A., Pessarakli M. (2010). A review on biological control of fungal plant pathogen using microbial antagonists. J. Biol. Sci..

[66] Bonaterra A., Badosa E., Daranas N., Francés J., Roselló G., Montesinos E. (2022). Bacteria as biological control agents of plant diseases. Microorganisms.

[67] Tsotetsi T., Nephali L., Malebe M., Tugizimana F. (2018). *Bacillus* for plant growth promotion and stress resilience: What have we learned?. Plants.

[68] Zeigler D.R., Perkins J.B. (2008). The genus *Bacillus*. Practical Handbook of Microbiology.

[69] Saxena A.K., Kumar M., Chakdar H., Anuroopa N., Bagyaraj D.J. (2019). *Bacillus* species in soil as a natural resource for plant health and nutrition. J. Appl. Microbiol..

[70] Piggot P., Hilbert D. (2004). Sporulation of *Bacillus subtilis*. Curr. Opin. Microbiol..

[71] Haque M.M., Mosharaf M.K., Khatun M., Haque M.A., Biswas M.S., Islam M.S., Islam M.M., Shozib H.B., Miah M.M.U., Molla A.H. (2020). Biofilm producing rhizobacteria with multiple plant growth-promoting traits promote growth of tomato under water-deficit stress. Front. Microbiol..

[72] Sansinenea E., Singh H., Keswani C., Reddy M., Sansinenea E., García-Estrada C. (2019). Bacillus spp.: As plant growth-promoting bacteria. Secondary Metabolites of Plant Growth Promoting Rhizomicroorganisms.

[73] Su Y., Liu C., Fang H., Zhang D. (2020). *Bacillus subtilis*: A universal cell factory for industry, agriculture, biomaterials and medicine. Microb. Cell Fact..

[74] Zhang N., Wang Z., Shao J., Xu Z., Liu Y., Xun W., Miao Y., Shen Q., Zhang R. (2023). Biocontrol mechanisms of *Bacillus*: Improving the efficiency of green agriculture. Microb. Biotechnol..

[75] Beneduzi A., Ambrosini A., Passaglia L.M. (2012). Plant growth-promoting Rhizobacteria (PGPR): Their potential as antagonists and biocontrol agents. Genet. Mol. Biol..

[76] Hashem A., Tabassum B., Abd_Allah E.F. (2019). *Bacillus subtilis*: A plant-growth promoting rhizobacterium that also impacts biotic stress. Saudi J. Biol. Sci..

[77] Xu M., Sheng J., Chen J., Men Y., Gan L., Guo S., Shen L. (2014). Bacterial community compositions of tomato (*Lycopersicum esculentum* Mill.) seeds and plant growth promoting activity of ACC deaminase producing *Bacillus subtilis* (HYT-12-1) on tomato seedlings. World J. Microbiol. Biotechnol..

[78] Jain S., Varma A., Choudhary D.K., Cruz C., Vishwakarma K., Choudhary D.K., Varma A. (2021). Perspectives on nitrogen-fixing *Bacillus* species. Soil Nitrogen Ecology.

[79] Pattnaik S., Mohapatra B., Gupta A. (2021). Plant growth-promoting microbe mediated uptake of essential nutrients (Fe, P, K) for crop stress management: Microbe–soil–plant continuum. Front. Agron..

[80] Berdi J. (2005). Bioactive microbial metabolites. J. Antibiot..

[81] Fira D., Dimkić I., Berić T., Lozo J., Stanković S. (2018). Biological control of plant pathogens by *Bacillus* species. J. Biotechnol..

[82] Caulier S., Nannan C., Gillis A., Licciardi F., Bragard C., Mahillon J. (2019). Overview of the antimicrobial compounds produced by members of the *Bacillus subtilis* group. Front. Microbiol..

[83] Epparti P., Eligar S.M., Sattur A., Kumar B.S.G., Halami P.M. (2022). Characterization of dual bacteriocins producing *Bacillus subtilis* SC3.7 isolated from fermented food. Food Sci. Technol..

[84] Salazar F., Ortiz A., Sansinenea E. (2017). Characterisation of two novel bacteriocin-like substances produced by *Bacillus amyloliquefaciens* ELI149 with broad-spectrum antimicrobial activity. J. Glob. Antimicrob. Resist..

[85] Schneider T., Müller A., Miess H., Gross H. (2014). Cyclic lipopeptides as antibacterial agents—Potent antibiotic activity mediated by intriguing mode of actions. Int. J. Med. Microbiol..

[86] Helmy N.M., Parang K. (2023). Cyclic peptides with antifungal properties derived from bacteria, fungi, plants, and synthetic sources. Pharmaceuticals.

[87] Gutiérrez-Chávez C., Benaud N., Ferrari B.C. (2021). The ecological roles of microbial lipopeptides: Where are we going?. Comput. Struct. Biotechnol. J..

[88] Köhl J., Kolnaar R., Ravensberg J. (2019). Mode of action of microbial biological control agents against plant diseases: Relevance beyond efficacy. Front. Plant Sci..

[89] Meena K.R., Kanwar S.S. (2015). Lipopeptides as the antifungal and antibacterial agents: Applications in food safety and therapeutics. BioMed Res. Int..

[90] Bouchard-Rochette M., Machrafi Y., Cossus L., Nguyen T.T.A., Antoun H., Droit A., Tweddell R.J. (2022). *Bacillus pumilus* PTB180 and *Bacillus subtilis* PTB185: Production of lipopeptides, antifungal activity, and biocontrol ability against *Botrytis cinerea*. Biol. Control.

[91] Vignesh M., Shankar S.R.M., Subramani N., VedhaHari B.N., Ramzadevi D. (2022). Study on spray-drying of *Bacillus velezensis* NKMV-3 strain, its formulation and bio efficacy against early blight of tomato. Biocatal. Agric. Biotechnol..

[92] Jia Q., Fan Y., Duan S., Qin Q., Ding Y., Yang M., Wang Y., Liu F., Wang C. (2023). Effects of *Bacillus amyloliquefaciens* XJ-BV2007 on growth of *Alternaria alternata* and production of tenuazonic acid. Toxins.

[93] Kang B.R., Park J.S., Jung W.J. (2020). Antifungal evaluation of fengycin isoforms isolated from *Bacillus amyloliquefaciens* PPL against *Fusarium oxysporum* f. sp. *lycopersici*. Microb. Pathog..

[94] Solanki M.K., Singh R.K., Srivastava S., Kumar S., Kashyup P.L., Srivastava A.K. (2013). Characterization of antagonistic-potential of two *Bacillus* strains and their biocontrol activity against *Rhizoctonia solani* in tomato. J. Basic Microbiol..

[95] Pei D., Zhang Q., Zhu X., Zhang L. (2023). Biological control of *Verticillium* wilt and growth promotion in tomato by rhizospheric soil-derived *Bacillus amyloliquefaciens* Oj-2.16. Pathogens.

[96] Im S.M., Yu N.H., Joen H.W., Kim S.O., Park H.W., Park A.R., Kim J.C. (2020). Biological control of tomato bacterial wilt by oxydifficidin and difficidin-producing *Bacillus methylotrophicus* DR-08. Pestic. Biochem. Physiol..

[97] Yuan J., Li B., Zhang N., Waseem R., Shen Q., Huang Q. (2012). Production of bacillomycin- and macrolactin-type antibiotics by *Bacillus amyloliquefaciens* NJN-6 for suppressing soilborne plant pathogens. J. Agric. Food Chem..

[98] Singh D., Devappa V., Yadav D.K. (2022). Suppression of tomato bacterial wilt incited by *Ralstonia pseudosolanacearum* using polyketide antibiotic-producing *Bacillus* spp. isolated from rhizospheric soil. Agriculture.

[99] Chen Q., Qiu Y., Yuan Y., Wang K., Wang H. (2022). Biocontrol activity and action mechanism of *Bacillus velezensis* strain SDTB038 against Fusarium crown and root rot of tomato. Front. Microbiol..

[100] Garcia-Rubio R., de Oliveira H.C., Rivera J., Trevijano-Contador N. (2020). The Fungal cell wall: *Candida*, *Cryptococcus*, and *Aspergillus* species. Front. Microbiol..

[101] Khalil M.S.M., Hassan M.H.A.R., Mahmoud A.F., Morsy K.M.M. (2022). Involvement of secondary metabolites and extracellular lytic enzymes produced by plant growth promoting rhizobacteria in inhibiting the soilborne pathogens in faba bean plants. J. Trop. Plant Pests Dis..

[102] Kumar M., Brar A., Yadav M., Chawade A., Vivekanand V., Pareek N. (2018). Chitinases—Potential candidates for enhanced plant resistance towards fungal pathogens. Agriculture.

[103] Ghasemi S., Ahmadian G., Jelodar N.B., Rahimian H., Ghandili S., Dehestani A., Shariati P. (2010). Antifungal chitinases from *Bacillus pumilus* SG2: Preliminary report. World J. Microbiol. Biotechnol..

[104] Kilani-Feki O., Khedher S.B., Dammak M., Kamoun A., Jabnoun-Khiareddine H., Daami-Remadi M., Tounsi S. (2016). Improvement of antifungal metabolites production by *Bacillus subtilis* V26 for biocontrol of tomato postharvest disease. Biol. Control.

[105] Diabankana R.G.C., Shulga E.U., Validov S.Z., Afordoanyi D.M. (2022). Genetic characteristics and enzymatic activities of *Bacillus velezensis* KS04AU as a stable biocontrol agent against phytopathogens. Int. J. Plant Biol..

[106] Rocha F.Y.O., de Oliveira C.M., da Silva P.R.A., de Melo L.H.V., do Carmo M.G.F., Baldani J.I. (2017). Taxonomical and functional characterization of *Bacillus* strains isolated from tomato plants and their biocontrol activity against races 1, 2 and 3 of *Fusarium oxysporum* f. sp. *lycopersici*. Appl. Soil Ecol..

[107] Dubinkina V., Fridman Y., Pandey P.P., Maslov S. (2019). Multistability and regime shifts in microbial communities explained by competition for essential nutrients. eLife.

[108] Lahlali R., Ezrari S., Radouane N., Kenfaoui J., Esmaeel Q., El Hamss H., Belabess Z., Barka E.A. (2022). Biological control of plant pathogens: A global perspective. Microorganisms.

[109] Radhakrishnan R., Hashem A., Abd_Allah E.F. (2017). *Bacillus*: A biological tool for crop improvement through bio-molecular changes in adverse environments. Front. Physiol..

[110] Mates A.P.K., Pontes N.C., Halfeld-Vieira B.A. (2019). *Bacillus velezensis* GF267 as a multi-site antagonist for the control of tomato bacterial spot. Biol. Control.

[111] Tan S., Jiang Y., Song S., Huang J., Ling N., Xu Y., Shen Q. (2013). Two *Bacillus amyloliquefaciens* strains isolated using the competitive tomato root enrichment method and their effects on suppressing *Ralstonia solanacearum* and promoting tomato plant growth. Crop Prot..

[112] Wu K., Su L., Fang Z., Yuan S., Wang L., Shen B., Shen Q. (2017). Competitive use of root exudates by *Bacillus* amyloliquefaciens with *Ralstonia solanacearum* decreases the pathogenic population density and effectively controls tomato bacterial wilt. Sci. Hortic..

[113] Khan A., Doshi H.V., Thakur M.C., Islam M., Rahman M., Pandey P., Jha C., Aeron A. (2016). Bacillus spp.: A prolific siderophore producer. Bacilli and Agrobiotechnology.

[114] Saha R., Saha N., Donofrio R.S., Bestervelt L.L. (2012). Microbial siderophores: A mini review. J. Basic Microbiol..

[115] Roskova Z., Skarohlid R., McGachy L. (2022). Siderophores: An alternative bioremediation strategy?. Sci. Total Environ..

[116] Kalam S., Basu A., Podile A.R. (2020). Functional and molecular characterization of plant growth promoting *Bacillus* isolates from tomato rhizosphere. Hellyon.

[117] Xu S.J., Park D.H., Kim J.Y., Kim B.S. (2016). Biological control of grey mold and growth promotion of tomato using *Bacillus* spp. isolated from soil. Trop. Plant Pathol..

[118] Dong H., Gao R., Dong Y., Yao Q., Zhu H. (2023). *Bacillus velezensis* RC116 inhibits the pathogens of bacterial wilt and fusarium wilt in tomato with multiple biocontrol traits. Int. J. Mol. Sci..

[119] Gautam S., Chauhan A., Sharma R., Sehgal R., Shirkot C.K. (2019). Potential of *Bacillus amyloliquefaciens* for biocontrol of bacterial canker of tomato incited by *Clavibacter michiganensis* ssp. *michiganensis*. Microb. Pathog..

[120] Schmidt R., Cordovez V., de Boer W., Raaijmakers J., Garbeva P. (2015). Volatile affairs in microbial interactions. ISME J..

[121] Gámez-Arcas S., Baroja-Fernández E., García-Gómez P., Muñoz F.J., Almagro G., Bahaji A., Sánchez-López Á.M., Pozueta-Romero J. (2022). Action mechanisms of small microbial volatile compounds in plants. J. Exp. Bot..

[122] Peñuelas J., Asensio D., Tholl D., Wenke K., Rosenkranz M., Piechulla B., Schnitzler J.P. (2014). Biogenic volatile emissions from the soil. Plant Cell Environ..

[123] Ramyabharathi S.A., Raguchander T. (2014). Mode of action of *Bacillus subtilis* EPCO16 against tomato Fusarium wilt. Biochem. Cell. Arch..

[124] Sachdev S., Singh R.P. (2018). Isolation, characterisation and screening of native microbial isolates for biocontrol of fungal pathogens of tomato. Clim. Chang. Environ. Sustain..

[125] Wang S., Hu T., Jiao Y., Wei J., Cao K. (2009). Isolation and characterization of *Bacillus subtilis* EB-28, an endophytic bacterium strain displaying biocontrol activity against *Botrytis cinerea* Pers. Front. Agric. China.

[126] Raza W., Wang J., Wu Y., Ling N., Wei Z., Huang Q., Shen Q. (2016). Effects of volatile organic compounds produced by *Bacillus amyloliquefaciens* on the growth and virulence traits of tomato bacterial wilt pathogen *Ralstonia solanacearum*. Appl. Microbiol. Biotechnol..

[127] Awan Z.A., Shoaib A., Schenk P.M., Ahmad A., Alansi S., Paray B.A. (2023). Antifungal potential of volatiles produced by *Bacillus subtilis* BS-01 against *Alternaria solani* in *Solanum lycopersicum*. Front. Plant Sci..

[128] Guo J., Xu Y., Liang S., Zhou Z., Zhang C., Li K., Peng X., Qin S., Xing K. (2023). Antifungal activity of volatile compounds from *Bacillus tequilensis* XK29 against *Botrytis cinerea* causing gray mold on cherry tomatoes. Postharvest Biol. Technol..

[129] Lee S.W., Lee S.H., Balaraju K., Park K.S., Nam K.W., Park J.W., Park K. (2014). Growth promotion and induced disease suppression of four vegetable crops by a selected plant growth-promoting rhizobacteria (PGPR) strain *Bacillus subtilis* 21-1 under two different soil conditions. Acta Physiol. Plant..

[130] Chowdappa P., Kumar S.P.M., Lakshmi M.J., Upreti K.K. (2013). Growth stimulation and induction of systemic resistance in tomato against early and late blight by *Bacillus subtilis* OTPB1 or *Trichoderma harzianum* OTPB3. Biol. Control.

[131] Farzand A., Moosa A., Zubair M., Khan A.R., Massawe V.C., Tahir H.A.S., Sheikh T.M.M., Ayaz M., Gao X. (2019). Suppression of *Sclerotinia sclerotiorum* by the induction of systemic resistance and regulation of antioxidant pathways in tomato using fengycin produced by *Bacillus amyloliquefaciens* FZB42. Biomolecules.

[132] Zhou L., Song C., Muñoz C., Kuipers O. (2021). *Bacillus cabrialesii* BH5 protects tomato plants against *Botrytis cinerea* by production of specific antifungal compounds. Front. Microbiol..

[133] Yan Y., Xu W., Hu Y., Tian R., Wang Z. (2022). *Bacillus velezensis* YYC promotes tomato growth and induces resistance against bacterial wilt. Biol. Control.

[134] Chandrasekaran M., Belachew S.T., Yoon E., Chun S.C. (2017). Expression of β-1,3-glucanase (*GLU*) and phenylalanine ammonia-lyase (*PAL*) genes and their enzymes in tomato plants induced after treatment with *Bacillus subtilis* CBR05 against *Xanthomonas campestris* pv. *vesicatoria*. J. Gen. Plant Pathol..

[135] Rashad Y.M., Abdalla S.A., Sleem M.M. (2022). Endophytic *Bacillus subtilis* SR22 triggers defense responses in tomato against rhizoctonia root rot. Plants.

[136] Van Loon L.C. (2007). Plant responses to plant growth-promoting rhizobacteria. Eur. J. Plant Pathol..

[137] Dimopoulou A., Theologidis I., Liebmann B., Kalantidis K., Vassilakos N., Skandalis N. (2019). *Bacillus amyloliquefaciens* MBI600 differentially induces tomato defense signaling pathways depending on plant part and dose of application. Sci. Rep..

[138] Appu M., Ramalingam P., Sathiyanarayanan A., Huang J. (2021). An overview of plant defense-related enzymes responses to biotic stresses. Plant Gene.

[139] Ongena M., Jourdan E., Adam A., Michel P., Brans A., Joris B., Arpigny J.L., Thonart P. (2007). Surfactin and fengycin lipopeptides of *Bacillus subtilis* as elicitors of induced systemic resistance in plants. Environ. Microbiol..

[140] Samaras A., Roumeliotis E., Ntasiou P., Karaoglanidis G. (2021). *Bacillus subtilis* mbi600 promotes growth of tomato plants and induces systemic resistance contributing to the control of soilborne pathogens. Plants.

[141] Awan Z.A., Shaaib A. (2019). Combating early blight infection by employing *Bacillus subtilis* in combination with plant fertilizers. Curr. Plant Biol..

[142] Kipngeno P., Losenge T., Maina N., Kahangi E., Juma P. (2015). Efficacy of *Bacillus subtilis* and *Trichoderma asperellum* against *Pythium aphanidermatum* in tomatoes. Biol. Control.

[143] Wu K., Fang Z., Wang L., Yuan S., Guo R., Shen B., Shen Q. (2016). Biological potential of bioorganic fertilizer fortified with bacterial antagonist for the control of tomato bacterial wilt and the promotion of crop yields. J. Microbiol. Biotechnol..

[144] Bahramisharif A., Rose L.E. (2019). Efficacy of biological agents and compost on growth and resistance of tomatoes to late blight. Planta.

[145] Ji X., Li J., Meng Z., Zhang S., Dong B., Qiao K. (2019). Synergistic effect of combined application of a new fungicide fluopimomide with a biocontrol agent *Bacillus methylotrophicus* TA-1 for management of gray mold in tomato. Plant Dis..

[146] Peng D., Luo K., Jiang H., Deng Y., Bai L., Zhou X. (2017). Combined use of Bacillus subtilis strain B-001 and bactericide for the control of tomato bacterial wilt. Pest Manag. Sci..

[147] Myresiotis C.K., Karaoglanidis G.S., Vryzas Z., Papadopoulou-Mourkidou E. (2012). Evaluation of plant-growth-promoting rhizobacteria, acibenzolar-S-methyl and hymexazol for integrated control of Fusarium crown and root rot on tomato. Pest Manag. Sci..

[148] Esquivel-Cervantes L.F., Tlapal-Bolaños B., Tovar-Pedraza J.M., Pérez-Hernández O., Leyva-Mir S.G., Camacho-Tapia M. (2022). Efficacy of biorational products for managing diseases of tomato in greenhouse production. Plants.

[149] Mousa M.A.A., Abo-Elyousr K.A.M., Abdel Alal A.M.K., Alshareef N.O. (2021). Management Fusarium wilt disease in tomato by combinations of *Bacillus amyloliquefaciens* and peppermint oil. Agronomy.

[150] Abdeljalil N.O.B., Vallance J., Gerbore J., Yacoub A., Daami-Remadi M., Rey P. (2021). Combining potential oomycete and bacterial biocontrol agents as a tool to fight tomato Rhizoctonia root rot. Biol. Control.

[151] Zhao X., Hendriks M., Deleu E., Spanoghe P., Höfte M., van Overbeek L., Uyttendaele M. (2023). Prevalence, attachment ability and strength of the biological control agent *Bacillus thuringiensis* on tomato. Food Microbiol..

[152] Chien Y.C., Huang C.H. (2020). Biocontrol of bacterial spot on tomato by foliar spray and growth medium application of *Bacillus amyloliquefaciens* and *Trichoderma asperellum*. Eur. J. Plant Pathol..

[153] Kulimushi S.M., Muiru W.M., Mutitu E.W. (2021). Potential of *Trichoderma* spp., *Bacillus subtilis* and *Pseudomonas fluorescens* in the management of early blight in tomato. Biocontrol Sci. Technol..

[154] Devi N.O., Tombisana Devi R.K., Debbarma M., Hajong M., Thokchom S. (2022). Effect of endophytic *Bacillus* and arbuscular mycorrhiza fungi (AMF) against Fusarium wilt of tomato caused by *Fusarium oxysporum* f. sp. *lycopersici*. Egypt. J. Biol. Pest Control.

[155] Sultana F., Hossain M.M. (2022). Assessing the potentials of bacterial antagonists for plant growth promotion, nutrient acquisition, and biological control of Southern blight disease in tomato. PLoS ONE.

[156] Elsayed T.R., Jacquiod S., Nour E.H., Sørensen S.J., Smalla K. (2020). Biocontrol of bacterial wilt disease through complex interaction between tomato plant, antagonists, the indigenous rhizosphere microbiota, and *Ralstonia solanacearum*. Front. Microbiol..

[157] Solanki M.K., Solanki A.C., Rai S., Srivastava S., Kashyap B.K., Divvela P.K., Kumar S., Yandigeri M.S., Kashyap P.L., Shrivastava A.K. (2022). Functional interplay between antagonistic bacteria and *Rhizoctonia solani* in the tomato plant rhizosphere. Front. Microbiol..

[158] Khalil M.E. (2019). Efficiency of *Trichoderma viride* and *Bacillus subtilis* as biocontrol agents against root rot caused by *Fusarium solani* in tomato. Egypt. J. Agric. Res..

